# A green and facile preparation approach, licochalcone A capped on hollow gold nanoparticles, for improving the solubility and dissolution of anticancer natural product

**DOI:** 10.18632/oncotarget.22387

**Published:** 2017-11-11

**Authors:** Yi-Wei Sun, Li-Hong Wang, Da-Li Meng, Xin Che

**Affiliations:** ^1^ School of Traditional Chinese Materia Medica, Key Laboratory of Structure-Based Drug Design and Discovery (Shenyang Pharmaceutical University), Ministry of Education, Shenyang 110016, PR China; ^2^ School of Pharmaceutical Engineering, Shenyang Pharmaceutical University, Shenyang 110016, PR China; ^3^ School of Pharmacy, Shenyang Pharmaceutical University, Shenyang 110016, PR China

**Keywords:** licochalcone A, hollow gold nanoparticles, solubility, dissolution, green method

## Abstract

This study described a valuable drug delivery system for poorly water-soluble anticancer naturalproduct, licochalcone A, isolated from *Glycyrrhiza inflata*, loaded on hollow gold nanoparticles by green method to improve solubility and dissolution and maintain its natural pharmacological property. Briefly, the formation of hollow gold nanoparticles involves three steps: preparing of silica nanospheres by Stober method, forming of a thick gold shell around the silica templates and etching of silica particles by HF solution. Hollow gold nanoparticles (HGNPs) and drug loaded hollow gold nanoparticles (L-HGNPs) displayed spherical structure and approximately 200nm in size observed by SEM, XRD, EDS and DSC analysis showed that HGNPs were gold hollow structure and crystalline form. The solubility in aqueous solution of licochalcone A was increased obviously to 488.9 μg/ml, compared with free drugs of 136.1 μg/ml. Another interesting finding is that near-infrared (NIR) irradiation increased the speed of solubility of licochalcone A in aqueous solutions, rather than quantity. In short, the method of nano-delivery system combined with poorly water-soluble drug to improve its solubility and dissolution is worth applying to other natural products in order to increase their opportunities in clinical applications.

## INTRODUCTION

It is generally accepted that chemotherapeutics, the most effective treatment for cancers, however, areless effect against those cancer cells which have developed simultaneously drug resistance, making thepatients suffering from such cancers extremely painful owing to seriously side effects [1]. With the development of traditional Chinese medicine, natural products were attached great attention in the world due to their characteristic biological activity, multi-targeted properties in multi-systemic diseases [2], green and natural resources with less side effects [3], such as vinblastine, vincristine [4], harringtonine and camptothecin [5]. Consequently, natural products have played a major role during the development of new drugs [6]. However, the existing natural products with high anticancer activity are still associated with various deficiencies and shortcomings, such as poor target selectivity, low yields as well as poor solubility [7–9], which obstruct their application in clinical treatment. Therefore, how to overcome these shortages has become a crucial subject to the researchers.

In this situation, optimization of the vehicles is necessary for TCM or the natural product. Nanotechnology, with high drug-loading capacity, high biocompatibility and biodegradability, are currently being widely used in the fields of delivery systems, in terms of increased solubility, improved bioavailability as well as target-rapid released for natural products [10, 11]. Concerning about the problem of chemical pollution of ethosome, liposome and niosome, inorganic-based nanoparticles as green and non-chemical additional carrier could reduce side effects as well as exhibit optical properties [12]. Among the various kinds of nanotechnology, gold nanoparticle, has obtained great interest in the fields of drug delivery, gene therapy and biomedical imaging [13–15]. Due to the advantages of facile surface modification, large load capability, outstanding stability and biocompatibility, it has excellent characteristics as a good vehicle for natural products to maintain their original biological activities [16–18]. In our previous study [19], the solubility and dissolution of the poor water-soluble xanthoceraside had been significantly improved after loaded onto the surface of gold nanoparticles, suggesting the applicability of gold nanoparticles in other natural products. More importantly, gold nanoparticles could absorb the specific frequency of light, such as near-infrared light, resulting in collective oscillation of electrons on the surface defined as surface plasmon resonance (SPR) [20, 21]. The high heat generated through specialized SPR properties could effectively ablate the cancerous cells [22], which make laser-induced photothermal ablation (PTA) widely used in anticancer therapy, consequently [23].

Licochalcone A, a natural chalcone extensively derived from the roots of *Glycyrrhiza uralensis* Fisch or the fruits and leaves of *Stauntonia brachyanthera* Hand-Mazz [24], has exhibited an array of pharmacological activities, such as anti-inflammatory [25], antioxidant and anticancer activity, especially in gastric, breast and cervical cancers [26–28]. However, the poor solubility and low bioavailability in aqueoussolution due to the lipophilic moiety in the structure have become the bottleneck for its development into a potential clinic candidate [29]. In this study, licochalcone A was successfully loaded onto hollow gold nanoparticles (L-HGNPs) by ultrasonic method, and revealed the influence of NIR irradiation on its solubility.

## RESULTS

### Structure identification

In the ^1^HNMR spectrum (300MHz, DMSO-*d*_6_), the trans-olefinic protons at *δ* 7.90 and 7.63 (each 1H, d, *J* = 15.6 Hz), the signals of AA'BB’ coupling system *δ* 7.98 and 6.89 (each 2H, d, *J* = 8.9 Hz), two singlet aromatic protons at *δ* 6.53 and 7.53 (each 1H, s), as well as the characteristic isopentenyl signals at *δ* 6.25 (1H, dd, *J* =10.3, 17.8 Hz), 4.96 (1H, m), 4.91 (1H, m), and 1.46 (6H, s) and a methoxy at *δ* 3.83 (3H, s) confirmed the structure of licochalcone A, which was proved by its ^13^C NMR (75 MHz, DMSO-*d*_6_) signals (*δ* 187.4, 161.9, 159.9, 158.4, 147.6, 138.8, 138.8, 138.8, 129.7, 127.8, 127.8, 117.8, 115.4, 115.4, 115.4, 113.6, 110.1, 100.1, 55.5, 40.4, 27.1, 27.1, respectively), and the reported data [40] (Table 1 and Supplementary Figures 1 and 2).

**Table 1:** Chemical shifts of licochalcone A

Position	*δ*H	*δ*C
1	113.5	
2	158.3	
3	100.1	6.53 (1H, s)
4	159.9	
5	126.7	
6	127.8	7.53 (1H, s)
*α*	117.8	7.63 (1H, d, *J*=15.6 Hz)
*β*	138.8	7.90 (1H, d, *J*=15.6 Hz)
C=O	187.4	
1′	129.7	
2′,6′	130.8	7.98 (2H, d, *J*=8.9 Hz)
3′,5′	115.4	6.89 (2H, d,*J*=8.9 Hz)
4′	161.8	
1′	40.3	
2′	147.6	6.25 (1H, dd, *J*=17.8,10.3 Hz)
3′	110.1	4.93 (1H, m) 4.96 (1H, m)
4′,5′	27.1	1.46 (6H, s)
-OCH_3_	55.5	3.83 (3H, s)

(^1^H: 600 MHz, ^13^C: 150 MHz in DMSO-*d*_6_)

### Characteristics of HGNPs and L-HGNPs

**Figure 1:**
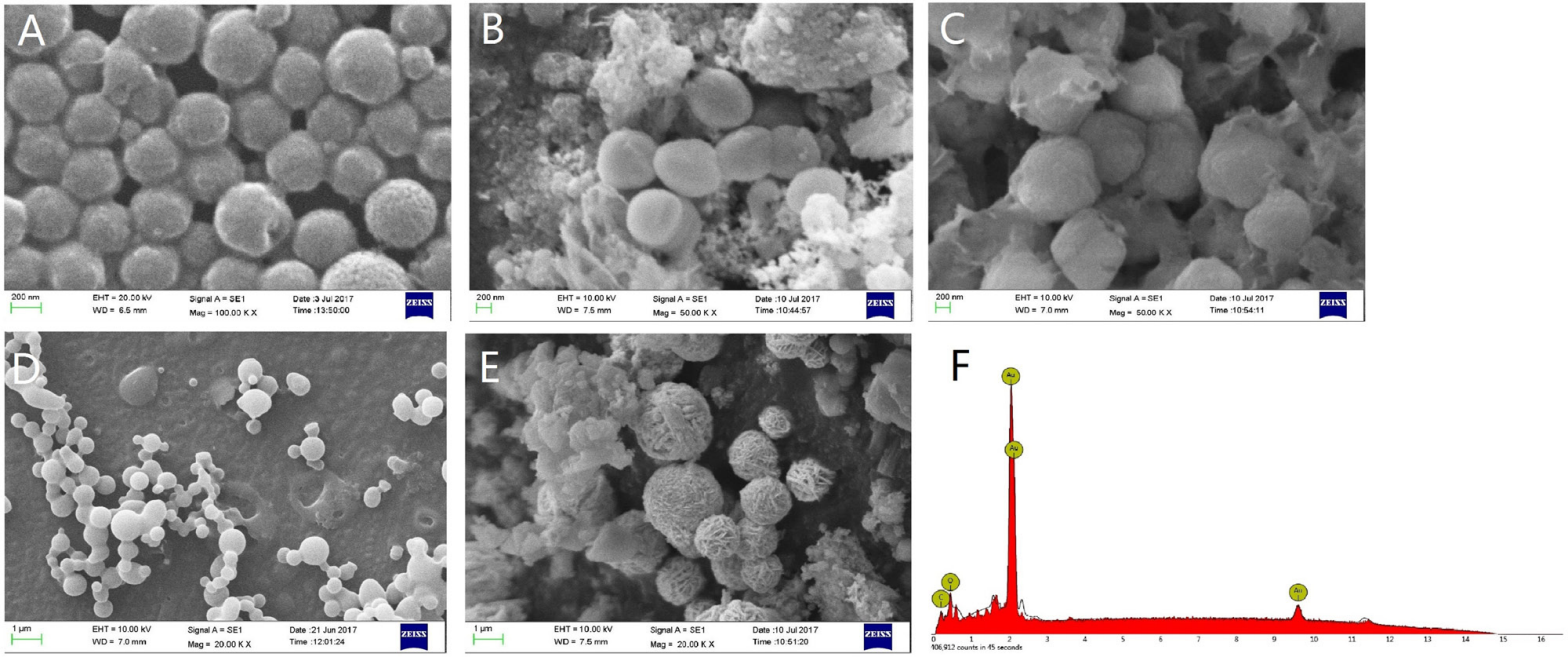
Representative of SEM images and EDS results SEM images of the HGNPs **(A)**. SEM images of licochalcone A/hollow gold nanoparticles at 0.25mg/mg **(B)**, 0.50mg/mg **(C)**, 1.00mg/mg **(D)**, 2.00mg/mg **(E)**. EDS pattern of the hollow nanostructures **(F)**.

SEM imaging showed that gold nanoparticles had typical diameters of 200 nm (Figure 1A) and the prepared nanostructures were hollow spherical gold shells. The EDS pattern of hollow gold nanoparticles (Figure 1F) indicated that hollow gold nanoparticles weremade up of over 99% gold element, and revealed the silica spheres template were etched completely by 2.5% HF solution with the structure of gold shell un-destroyed. As SEM images described, it could be obviously observed that the wall of hollow gold nanoparticles was thickened after licochalcone A was loaded onto HGNPs by ultrasonic effect and the surface of gold nanoparticles was filled of drugs. When the ratios of drug/HGNPs were at 0.25 mg/mg and 0.5 mg/mg (Figure 1B and 1C), licochalcone A would be attached on the surface of gold nanoparticles homogeneously and tightly like a membranous state, formed as pherical appearance of diameters of 300 nm nanoparticle with smooth surface instead of its own tiny gold particles. However, when the ratio increased to 1.0 mg/mg (Figure 1D), the shell became an opaque state and gold nanoparticles would be transformed into aggregation and adhesion due to drug overload without diameter increased. When the ratio was further increased to 2.0 mg/mg (Figure 1E), licochalcone A was not only loaded on the wall of hollow gold nanoparticles by a stable structure, but also formed the original drug's crystal out of gold nanoparticles and the diameter sharply increased for about 800nm. Therefore, the prepared HGNPs had great loadable area that the suitable ratio of licochalcone A/HGNPs was about 0.50 mg/mg in the following experiments.

**Figure 2:**
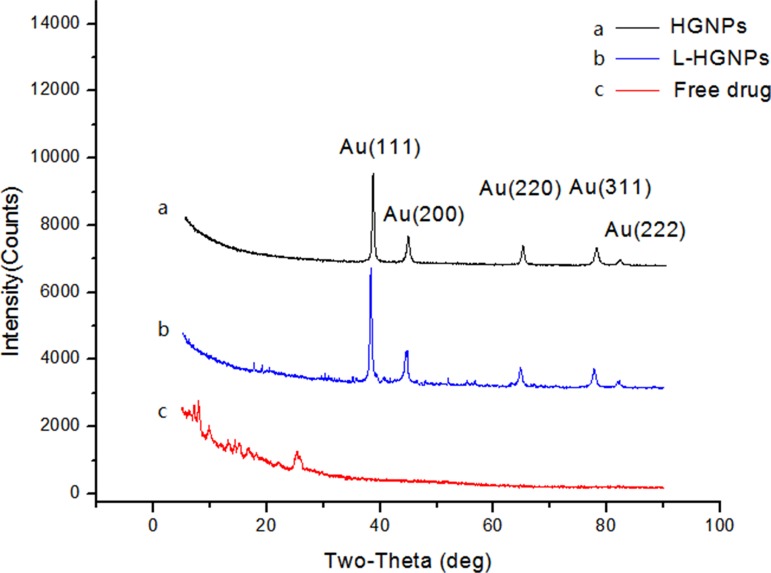
XRD images of hollow gold nanoparticles, L-HGNPs and free drug HGNPs (black), L-HGNPs (0.5mg/mg, blue) and free drug (red).

The crystal state of HGNPs, L-HGNPs and free licochalcone A were further confirmed by X-ray diffraction (Figure 2). The diffraction pattern for the HGNPs have five peaks at 38.18°, 44.45°, 64.64°, 77.62° and 81.77°, corresponding to the (111), (200), (220), (311) and (222) planes of the face centered cubic structure of gold [37], respectively (same as JCPDS 04-0784). Notably, the entirely different results between free drugs and L-HGNPs indicated that licochalcone A had lost its crystal state and integrated with gold nanoparticles.

**Figure 3:**
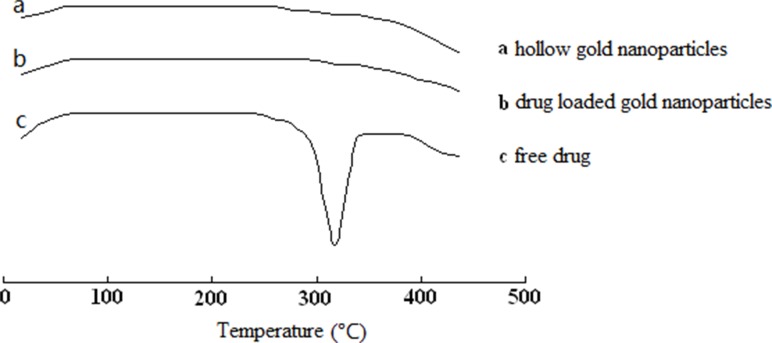
DSC thermogram of hollow gold nanoparticles **(A)**, drug loaded gold nanoparticles **(B)** and free drug **(C)**.

Additionally, Figure 3 illustrated the DSC thermograms of HGNPs, L-HGNPs and free licochalcone A. The pure drug showed a sharp endothermic peak at 308.2 °C, indicating the melting of the licochalcone A crystal. While, when the drug was loaded onto gold nanoparticles, the sharp peak disappeared owing to the presence of an amorphous state of licochalcone A.

### Solubility test

The effect of hollow gold nanoparticles on the solubility of licochalcone A was evaluated by using HPLC analyses. In the solubility tests, the concentrations of licochalcone A dissolved in aqueous was 136.1 μg/ml, while a sharp increased to 488.9 μg/ml when loaded on the hollow gold nanoparticles indicated that this system can significantly improved the solubility of poorly soluble drugs in water.

**Figure 4:**
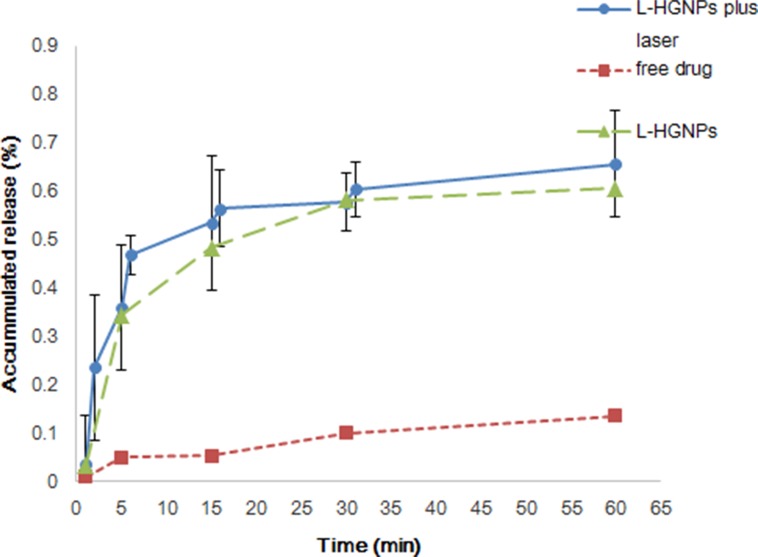
Dissolution profiles of free drugs and L-HGNPs with and without NIR laser irradiation The ratio of licochalcone A/HGNPs is 0.5mg/mg. At the designed intervals (1, 5, 15, 30, 60 min), the concentration of licochalcone A in the dissolution medium was analyzed by HPLC. In L-HGNPs plus laser group, the outpower of NIR light was 2 W for 1 min and then analyzed the concentration of licochalcone A at 1, 2, 5, 6, 15, 16, 30, 31, 60 min. The value of L-HGNPs with and without NIR laser irradiation were expressed as mean ± standard deviation (n = 3).

From our experiment results (Figure 4), the dissolution of pure drug was significantly lower than L-HGNPs, both of them were drawn as a smooth curve. However, on the laser group, there were more drugs dissolved in water after each time of irradiation, especially the first NIR irradiation which had a burst raised for about 23.5% of accumulated release, led to a broken line. After stirred for 3 h, the total amount of cumulative dissolution of NIR group was basically the same with non-NIR which implied that L-HGNPs after NIR laser excitation increased the dissolving rate instead of dissolving amount.

## DISCUSSION

Flavonoids, as the primarily class of natural products exist in herbal medicine, possess diverse bioactivities against cancer, infection and inflammation [30]. Chalcones is a special flavonoid that consist an open-chain of α, β-unsaturated carbonyl system joined with two aryl rings. These compounds with widespread distribution are reported to exhibit several pharmacological activities, including antiparasitic, antibacterial, antifungal, anticancer, nitric oxide inhibition and anti-inflammatory effects. Due to their structural diversity, some lead chalcones from both natural products and synthesis have been focused on molecular targets and cancer prevention and therapy [31]. Our model drug, licochalcone A, is receiving great attention in pharmacology for the remarkable and multifarious bioactivity, such as anti-inflammatory, antibacterial, antifungal, antiviral, antitumor as well as antiparasitic activity.

Unfortunately, this compound is hard to dissolve in water, which makes bioavailability extremely disappointing [32]. Hence, many solutions have been directed toward overcoming solubility and dissolution issues such as nanoemulsion technology, β-cyclodextrin encapsulation and phospholipid/bile salt micelles [33–36]. All the methods are currently applied as carriers for drug solubilizing. However, they all introduced extra solvents or organic materials in the process of synthesis more or less, resulting in unpredictable biological activity and effects. Furthermore, HGNPs could be administrated conveniently through intravenous and oral, without toxic effects in *vivo* experiment [42]. In our previous study, a system of drugs loaded on hollow gold nanoparticle by green ultrasonic method was successfully developed. It was a method of non-chemical pollution as well as kept the original bioactivity of drug for improving the dissolution. More importantly, it has unique optical and photothermal properties compared to other organic vehicle. On the basic of these, an efficient release system of L-HGNPs in aqueous solution was designed here, with NIR laser irradiation as the external stimulus, which could be potentially applied for improving the solubility and dissolution of poorly water-soluble anticancer natural product.

Compared with most research of solubility enhancing strategies, a carrier of green, nonorganic addition was prepared and applied in this paper. It is well known that one of the possible approaches for increasing the solubility and dissolution rate of a drug is to convert it from the crystalline to the amorphous form [38]. The results of SEM images and EDS experiment provided substantial evidences that our model drug at the appropriate concentration, had loaded on the surface of HGNPs with an amorphous form instead of crystalline nature without any chemical interaction. While, once it's overloaded, it would present the original crystal outside of HGNPs. In Figure 2, compared with free drugs in XRD, the disappearance of its own unique diffraction peaks in L-HGNPs indicated the change of crystal structure. Moreover, in the DSC test, an endothermic peak due to the melting of licochalcone A crystal, as well as the disappearance of the sharp peak in L-HGNPs confirmed the transformation of licochalcone A from crystal to amorphous state. Therefore, at the suitable ratio of licochalcone A/HGNPs (0.50 mg/mg), L-HGNPs showed 2.8-fold increment in the dissolution rate compared with pure drug due to this kind of crystalline transformation. This phenomenon is understandable because the amorphous form possesses higher saturation solubility than their crystalline form [39]. The present results are consistent with those reported in our earlier work. Herein, on the basis of surface plasmon resonance (SPR) characteristic, the dissolving rate had an explosive increase after first NIR irradiation. This is because the gold nanoparticles convert light energy into heat energy and the temperature of the solution increases, resulting in an increase in solubility of licochalcone A in aqueous solution. Therefore, HGNPs can be successfully utilized to improve the solubility and the dissolution rate of licochalcone A. This method can provide an efficient and green solution to improve solubility of poor water-soluble natural products in clinical treatment. Furthermore, the addition of NIR irradiation can sharply promote the dissolution of drugs because of the relative high temperature generated from localized surface plasmon resonance (LSPR) effect [41].

## MATERIALS AND METHODS

### Chemicals and materials

Chloroauric acid was purchased from Energy Chemical Corporation. Ammonium hydroxide (25%), potassium carbonate, tetraethylorthosilicate (TEOS) (99%), ethanol absolute, hydrofluoric acid (40%), (3-aminopropyl) triethoxysilane (APTES), trisodium citrate dehydrate, sodium borohydride (98%), formaldehyde solution (37%) were purchased from Sinopharm Chemical RagentCorporation. 3-Aminopropyltriethoxysilane and deionized water was purchased from Aladdin Industial Corporation.

### Preparation of licochalcone A

**Figure 5:**
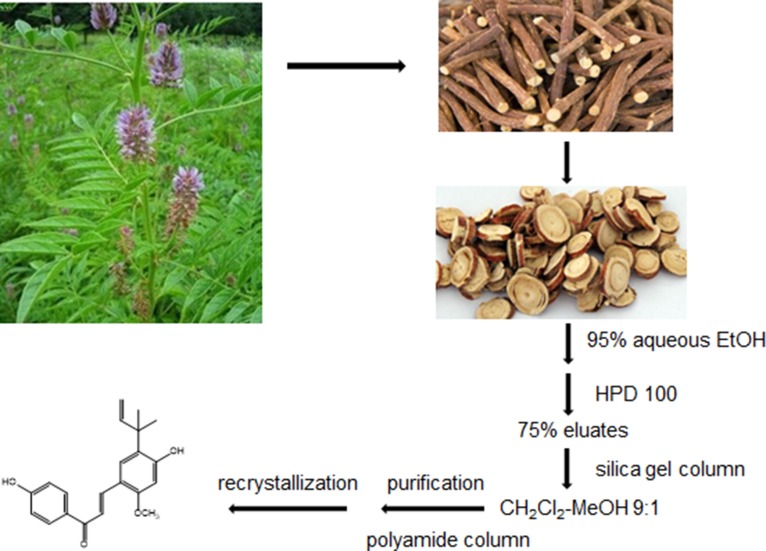
Methods of extraction and isolation of licochalcone A

The air-dried *G.inflata*, (10.0 kg) were extracted with 95% EtOH (160 L) under reflux for 2 h. After evaporation of the EtOH extracts *in vacuo*, the resultant residues (1.8 kg) were suspended in water and subjected to macroporous resin (HPD 100) to elute sequentially with H_2_O, 75%, 95%EtOH, respectively. The 75% eluates (120 g) were chromatographed on silica gel column (1000 mm×100 mm i.d.) using a gradient CH_2_Cl_2_-MeOH system and polyamide column chromatography with MeOH-H_2_O system as mobile phase, respectively. The licochalcone A was finally isolated from the fraction MeOH-H_2_O at 6:4 after recrystallization (Figure 5).

### Synthesis of hollow gold nanoparticles and drugs loading

Hollow gold nanoparticles were synthesized by our previous study. Briefly, prepared monodispersed silica spheres as Stober method described. Then, chloroauric acid was reduced by trisodium citrate and NaBH_4_ to obtain gold nanospheres (red colored solution). After the seeding step and the shell growth step, the obtained dark blue solution was centrifuged and redispersed in deionized water. The last step was to etch the silica templates to form hollow structure by HF solution, it changed from dark blue to light blue, indicating the formation of hollow structure. The hollow gold nanoparticles were collected by centrifugation, eventually.

For drugs loading, free licochalcone A dissolved in methanol and mixed with hollow gold nanoparticles with ultrasonic effect and the obtained L-HGNPs were collected by centrifugation at 12,000 rpm for 20 min and washed by methanol. The resulting L-HGNPs were purified by repeated centrifugation and washing steps. After that, methanol was evaporated in vacuum and licochalcone A were successfully loaded to the hollow gold nanoparticles with different drug-loading rates. Characterization

Different techniques were used to characterize prepared HGNPs and L-HGNPs. The shape and morphology were tested by scanning electron microscopy (SEM; ZEISS ULTRA 55, Germany) using an Ultra Plus field emission microscope operated at 20 kV. The crystal structure of the synthesized samples was characterized using an X-ray diffractometer (XRD; Empyrean PANalytical, Netherlands). Thermograms of HGNPs, L-HGNPs and free drugs were recorded by a Shimadzu calorimeter (Model DSC-60, Japan) under an inert atmosphere and loaded into an aluminum crucible, with a scanning rate of 10 °C/min over a temperature range between 30 and 430 °C.

### Solubility test

Licochalcone A dissolved in methanol was measured by using YMC-ODS-C18 Pack column (250mm×4.60mm) at the flow rate 1 ml/min at 25°C, and the mobile phase was methanol : water=75:25, v/v. Detection was performed at 210 nm and a sharp peak was obtained for licochalcone A at a retention time at about 15.7 min. Each sample was injected in the volume of 10 μl. A standard curve was established for licochalcone A (dissolved in methanol) at concentrations of 2, 1.5, 1, 0.75, 0.5, 0.25 mg/ml, respectively. All experiments were repeated three times. The linear regression equation was y = 969,906 x + 6547, R^2^ = 0.9995, (0.25 mg/ml≤x≤2 mg/ml) with concentration as abscissa and peak area as ordinate.

For solubility text by HPLC method, 2 mg of licochalcone A was dissolved in methanol and mixed with 1mg HGNPs. After methanol evaporation, 2 ml water was added and slowly stirred for 3 h at room temperature. After centrifugation, the supernatant was measured for their peak areas compared with 1 mg free drugs stirred in 2 ml water and operated ibid.

For the dissolution text, 0.5 mg of licochalcone A was used in free drug group and 1 mg licochalcone A mixed with 2 mg HGNPs were divided into NIR and non-NIR group. In NIR group, the samples were irradiated after each sampling by 808nm near-infrared light at 2.0 W for 1min. Each of group was added into 2 ml water and the concentration of licochalcone A in the dissolution medium obtained by centrifugation was analyzed by HPLC at the designed intervals1, 5, 15, 30, 60 min for non-NIR group and 1, 2, 5, 6, 15, 16, 30, 31, 60 min for NIR group.

## SUPPLEMENTARY FIGURES


