# Supplementary material for: A green and facile preparation approach, licochalcone A capped on hollow gold nanoparticles, for improving the solubility and dissolution of anticancer natural product
Source: Oncotarget. 2017 Nov 11;8(62):105673–81. doi: 10.18632/oncotarget.22387 (PMC5739669; doi:10.18632/oncotarget.22387)
Supplement: Supplementary file 1 [file oncotarget-08-105673-s001.pdf]

A green and facile preparation approach, licochalcone A capped on hollow gold nanoparticles, for improving the solubility and dissolution of anticancer natural product

SUPPLEMENTARY MATERIALS

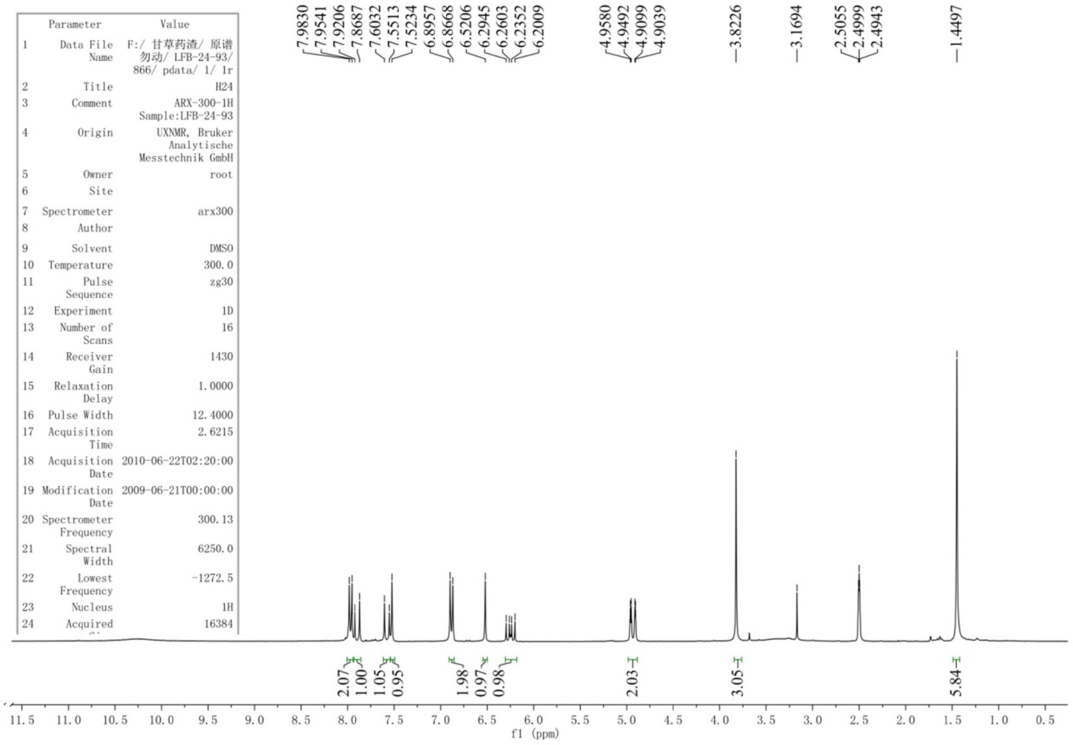

Supplementary Figure 1: ^{1}H-NMR Spectrum of licochalcone A.

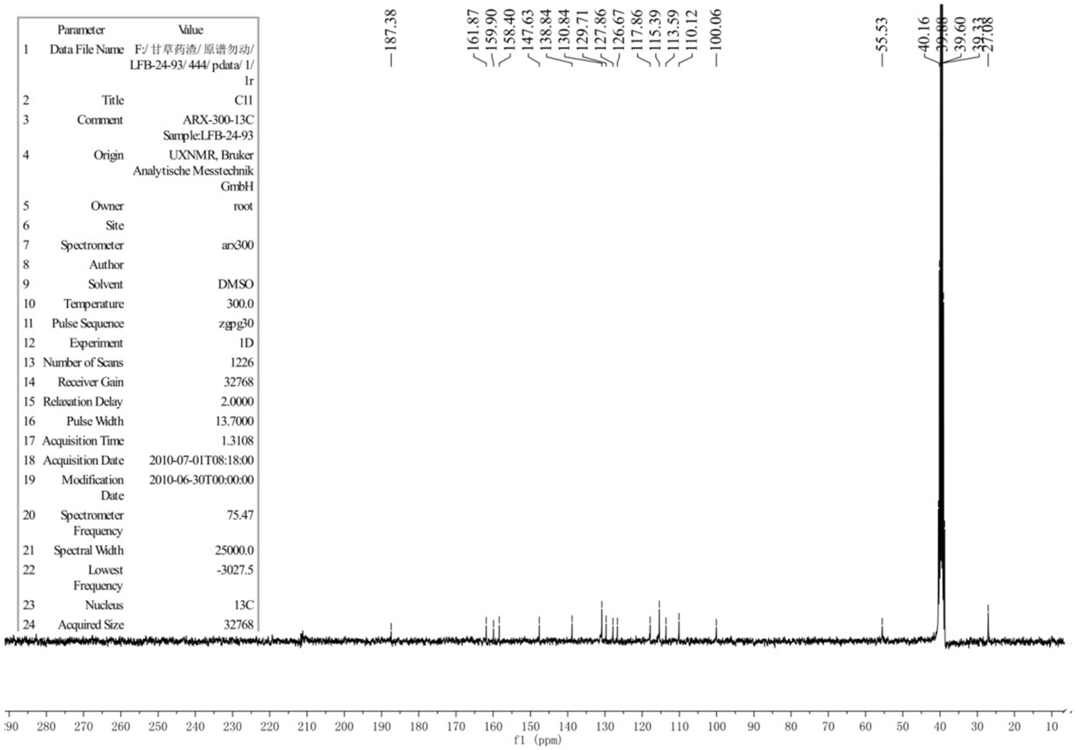

Supplementary Figure 2: ^{13}C-NMR Spectrum of licochalcone A.
